# Niche and local geography shape the pangenome of wastewater- and livestock-associated Enterobacteriaceae

**DOI:** 10.1126/sciadv.abe3868

**Published:** 2021-04-09

**Authors:** Liam P. Shaw, Kevin K. Chau, James Kavanagh, Manal AbuOun, Emma Stubberfield, H. Soon Gweon, Leanne Barker, Gillian Rodger, Mike J. Bowes, Alasdair T. M. Hubbard, Hayleah Pickford, Jeremy Swann, Daniel Gilson, Richard P. Smith, Sarah J. Hoosdally, Robert Sebra, Howard Brett, Tim E. A. Peto, Mark J. Bailey, Derrick W. Crook, Daniel S. Read, Muna F. Anjum, A. Sarah Walker, Nicole Stoesser, REHAB consortium

**Affiliations:** 1 Nuffield Department of Medicine, John Radcliffe Hospital, University of Oxford, Oxford OX3 9DU, UK.; 2 Department of Bacteriology, Animal and Plant Health Agency (APHA), Woodham Lane, Addlestone, Surrey KT15 3NB, UK.; 3 UK Centre for Ecology & Hydrology (UKCEH), Benson Lane, Crowmarsh Gifford, Wallingford OX10 8BB, UK.; 4 School of Biological Sciences, University of Reading, Reading RG6 6AS, UK.; 5 NIHR Oxford Biomedical Research Centre, John Radcliffe Hospital, Oxford OX3 9DU, UK.; 6 Department of Tropical Disease Biology, Liverpool School of Tropical Medicine, Pembroke Place, Liverpool L3 5QA, UK.; 7 NIHR Health Protection Research Unit in Healthcare Associated Infections and Antimicrobial Resistance at University of Oxford in partnership with Public Health England, Oxford OX4 9DU, UK.; 8 Department of Epidemiological Sciences, The Animal and Plant Health Agency (APHA), Woodham Lane, Addlestone, Surrey KT15 3NB, UK.; 9 Department of Genetics and Genomic Sciences, Icahn School of Medicine at Mount Sinai, New York, NY 10029, USA.; 10 Sema4, a Mount Sinai venture, 333 Ludlow Street, North Tower, 8th floor, Stamford, CT 06902, USA.; 11 Thames Water Utilities, Clearwater Court, Vastern Road, Reading RG1 8DB, UK.

## Abstract

*Escherichia coli* and other Enterobacteriaceae are diverse species with “open” pangenomes, where genes move intra- and interspecies via horizontal gene transfer. However, most analyses focus on clinical isolates. The pangenome dynamics of natural populations remain understudied, despite their suggested role as reservoirs for antimicrobial resistance (AMR) genes. Here, we analyze near-complete genomes for 827 Enterobacteriaceae (553 *Escherichia* and 274 non-*Escherichia* spp.) with 2292 circularized plasmids in total, collected from 19 locations (livestock farms and wastewater treatment works in the United Kingdom) within a 30-km radius at three time points over a year. We find different dynamics for chromosomal and plasmid-borne genes. Plasmids have a higher burden of AMR genes and insertion sequences, and AMR-gene-carrying plasmids show evidence of being under stronger selective pressure. Environmental niche and local geography both play a role in shaping plasmid dynamics. Our results highlight the importance of local strategies for controlling the spread of AMR.

## Teaser

The pangenome of *Escherichia coli* from nearby farms shows how livestock host and local geography shape bacterial populations.

## INTRODUCTION

Enterobacteriaceae are a family of Gram-negative bacteria that can cause clinical infections (*1*, *2*) and persist environmentally (*3*, *4*) across diverse environmental niches (*5*). Antimicrobial resistance (AMR) in Enterobacteriaceae has emerged as a major problem in the past decade (*6*, *7*). Dissemination of AMR genes often occurs via mobile genetic elements (MGEs), which can transfer genes within and between species both locally (*8*) and globally (*9*). Freshwater-, wastewater-, and livestock-associated strains of Enterobacteriaceae have been proposed as reservoirs for AMR genes in clinical isolates (*10*–*13*), but the links between these remain cryptic (*14*).

Species within Enterobacteriaceae are well-known examples of “open” pangenomes (*15*, *16*) containing substantial genetic diversity, with movement of genes via horizontal gene transfer (HGT) (*17*). Current understanding of the ecology and evolution of pangenomes is incomplete (*18*), with ongoing debate about the roles of niche adaptation and selection (*19*–*22*). However, published Enterobacteriaceae genomes are biased toward clinical isolates (*23*, *24*), and sampling frames reflecting truly interlinked communities are limited. Much remains unknown about the population genetics of Enterobacteriaceae (*25*) and the role of plasmids in nonclinical contexts (*26*).

Genomic studies of Enterobacteriaceae have predominantly used short-read whole-genome sequencing. AMR genes and their flanking regions are frequently fragmented in short-read assemblies due to repetitive elements and structural rearrangements (*8*, *27*). Combining short and long reads (“hybrid assembly”) produces complete, high-quality genomes (*28*), allowing accurate structural resolution. Here, we report a study of the multispecies pangenome within nonclinical Enterobacteriaceae using hybrid sequencing. We used hybrid assembly of 827 sympatric Enterobacteriaceae (including *Citrobacter*, *Enterobacter*, *Escherichia*, and *Klebsiella* spp.) to characterize the pangenome of these genera, considering both niche [cattle, pig, sheep, or wastewater treatment work (WwTW) associated] and geography (sampling location).

## RESULTS

### A diverse collection of complete genomes from livestock and water-borne niches

**Fig. 1:**
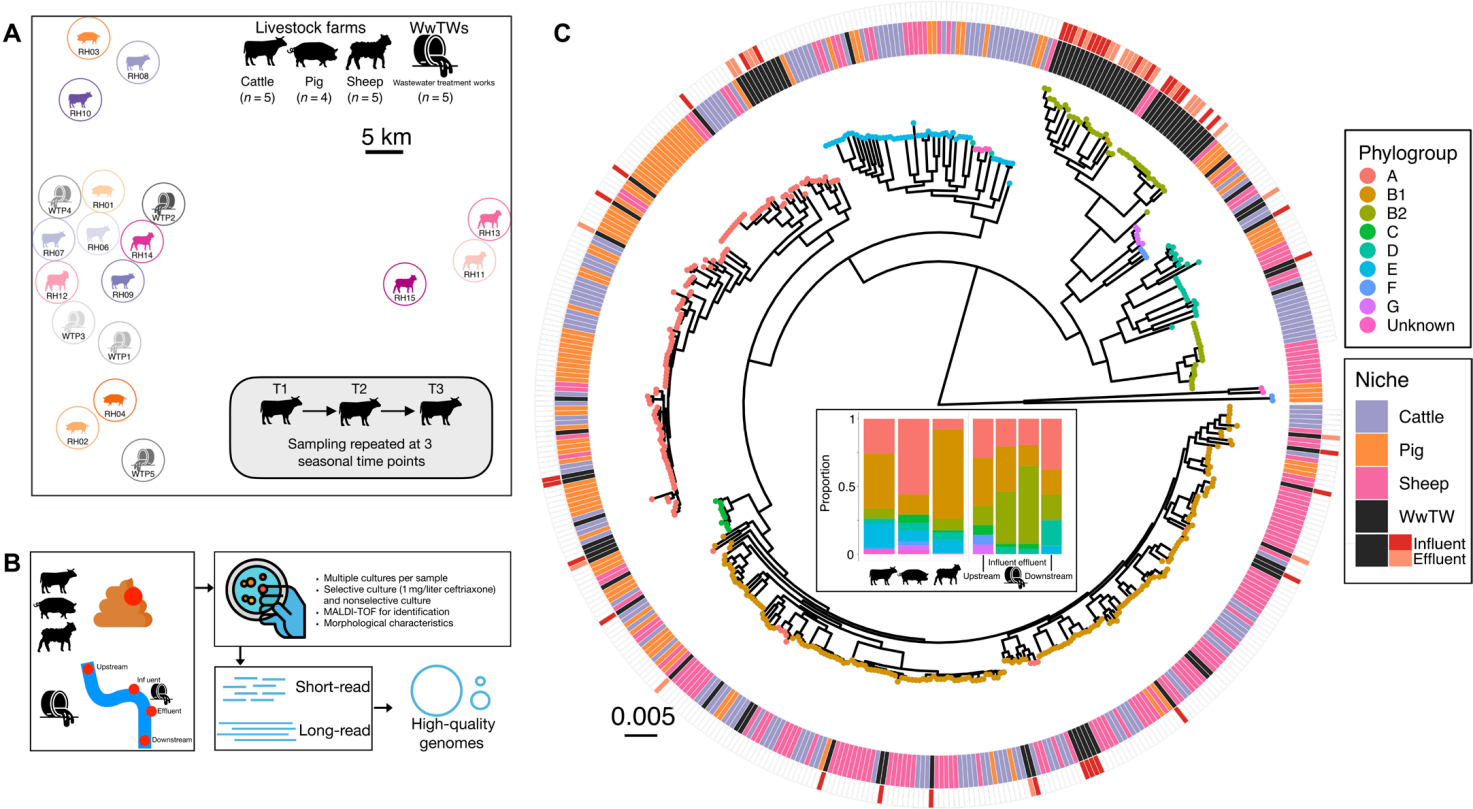
Overview of the diverse *Escherichia coli* isolates in this study. (**A**) Relative sampling locations of the farms (cattle, pig, and sheep) and wastewater treatment plants (WwTWs) in this study, sampled at three different TPs. (**B**) Schematic illustration of the sampling, culture, and sequencing workflow, resulting in high-quality genome assemblies with a median of one circularized chromosome and two circularized plasmids per assembly. (**C**) Mid-point rooted core genome phylogeny of *E. coli* isolates (*n* = 488), with tips colored by phylogroup and ring colors showing sampling niche. Inset panel at center of phylogeny shows phylogroup abundances (as proportion of isolates) from different sampling niches.

We collected samples from 19 locations ≥5 km apart (maximum distance: 60 km) in South Central England (United Kingdom) in 2017, namely, 14 livestock farms (four pig, five cattle, and five sheep) and water sources around five WwTWs over three seasonal time points (TPs) (Fig. 1A). We pooled samples within each location and TP and then cultured isolates from these pooled samples. A selected subset of 832 of 2098 cultured isolates underwent short- and long-read sequencing and hybrid genome assembly (Fig. 1B; see Materials and Methods), resulting in 827 high-quality genomes (table S1; *n* = 495 from livestock farms, *n* = 332 from WwTWs), from four genera: *Citrobacter* (*n* = 127), *Enterobacter* (*n* = 71), *Escherichia* (*n* = 553), and *Klebsiella* (*n* = 76). Most farm isolates were *Escherichia* spp. (451 of 495, 91.1%), with WwTW isolates having roughly even proportions of genera (fig. S1). Isolates contained a median of one AMR gene (range: 0 to 23), with variation by genus: *Klebsiella* isolates carried a median of 4 (range: 1 to 18).

Isolates were highly diverse, containing diversity not present in published genomes (fig. S2). *Escherichia* diversity included all main *E. coli* phylogroups (Fig. 1C), as well as 53 *Escherichia fergusonii*, and 13 isolates from clades I, II, III, and V (table S1). Phylogroup B2 was strongly associated with WwTWs compared with livestock (34.3% versus 5.3% of *Escherichia* isolates in each category respectively; χ^2^ = 70.4, *P* < 0.001), particularly in influent and effluent samples (Fig. 1C). This observation is in line with Ludden *et al.* (*29*), who found that phylogroup B2 was rare in livestock-associated *E. coli* sampled in England (4 of 431 genomes). Pigs had a greater proportion of phylogroup A isolates (Fig. 1C). Of 187 identified *E. coli* multilocus sequence types (STs), 56.1% (105 of 187) were seen only once, similar to the 61% observed by Touchon *et al.* (*30*) in a study of nonclinical *E. coli*. Only 12 *Escherichia* STs were seen in both livestock and WwTW isolates, with phylogroup B1 as the most represented (5 of 12 STs). ST10 was the most prevalent ST (*n* = 45), seen in 10 of 14 farms and 3 of 5 WwTWs. This is in agreement with recent studies in England: Ludden *et al.* (*29*) also found ST10 to be the most prevalent *E. coli* ST in livestock-associated isolates, and AbuOun *et al.* (*13*) found that the ST10 clonal complex was the most prevalent in isolates from pig farms. Our observations and good agreement with recent work in this setting suggest that our dataset, although sampled from a relatively small geographical region, is representative of broader nonclinical *E. coli* populations.

Considering only livestock *E. coli* isolates, over time, there was a persistent phylogroup signature of both livestock host and farm, with individual farm explaining slightly more variance than livestock type (*R*^2^ = 28.1% versus 25.8%; fig. S3). In other words, between-farm differences in *E. coli* phylogroup abundance were of the same order as differences between livestock species. However, livestock type explained less variance for STs than phylogroups (*R*^2^ = 8.5%), with only 39 of 131 STs (29.8%) seen on more than one farm. This suggests that associations between livestock species and *E. coli* population structure are more important at the higher level of phylogroup, and clear associations between ST and livestock species are not the norm. There were only 26 instances where an *E. coli* ST was observed over time on the same farm (involving 16 STs), and most of these (22 of 26 instances, 12 of 16 STs) were STs also seen across farms (fig. S4); these could therefore represent more generalist STs.

However, STs can encompass large genomic variation. We therefore also considered *E. coli* strain clusters using a core genome distance of <100 single-nucleotide variants (SNVs) (maximal diversity observed across sampled *E. coli*: 211,251 SNVs; median pairwise distance: 46,144 SNVs). There were 280 isolate pairs with <100 SNVs, of which 181 (64.6%) were isolates cultured from the same pooled sample (i.e., same farm and same TP) (fig. S5A). Overall, 10.5% of all isolate pairs from the same pooled sample had <100 SNVs between them, compared with 1.4% (*n* = 52) of isolate pairs from different TPs on the same farm and 0.2% (*n* = 44) between different farms of the same animal (fig. S5B). Of the latter, 41 of 44 were between cattle farms, and 36 involved a single cattle farm (RH06). There were only three isolate pairs with <100 SNVs between farms of different animals (fig. S5A). All of these were between farms in close geographic proximity (two instances from pig farm RH03 and cattle farm RH10, and one instance from cattle farm RH07 and sheep farm RH12; see Fig. 1A for distances), suggesting local strain movement. There were no isolate pairs with <100 SNVs between WwTW and livestock niches, and only three isolate pairs occurred across TPs at WwTWs (all at a single WwTW).

Together, these results indicate that different livestock hosts have a stable balance of *E. coli* phylogroups and that each farm setting can harbor substantial strain-level diversity, which, in our dataset, is unique to that farm and can persist over time. In contrast, isolates from locations proximal to WwTWs do not have a stable population of strains, reflecting the more transitory nature of this setting. We did not recover any transmission links at the strain level between WwTWs and livestock, although the vast diversity of natural *E. coli* populations means this should not be interpreted as evidence that these links do not exist.

### Plasmid gene repertoires are linked to genus and niche

We recovered 2292 circularized plasmids across all Enterobacteriaceae, ranging in size from 1240 bp to 824 kbp (median: 43 kbp; table S2). There were 297 of 2292 (13.0%) with no identifiable plasmid replicon, and most of these were from WwTW isolates (192 of 297, 64.6%). Multiple replicons were carried by 723 of 2292 (31.5%), and these plasmids tended to be larger (median length: 106,811 bp versus 6669 bp for single replicon plasmids). Of *E. coli* isolates with complete genomes, over two thirds (70.4%, 245 of 348) carried a plasmid with an IncFII replicon. Forty-three percent of circularized plasmids (986 of 2293) had at least one match with >99% identity to other publicly available plasmid sequences (fig. S2B). However, 12.3% (282 of 2293) had a top identity score of <95% to a previous known sequence (fig. S2B), and 17 plasmids with no match were identified, suggesting that sampling recovered previously undescribed plasmid diversity. We grouped circularized plasmids into 611 distinct plasmid clusters using alignment-free distances (see Materials and Methods), which closely matched their gene content (fig. S6A). A recent analysis by Redondo-Salvo *et al.* (*31*), clustering over 10,000 plasmids from prokaryotes using average nucleotide identity, found that plasmids within the same cluster contained a common genomic backbone. We also found that the synteny of shared genes was strongly conserved within plasmid clusters (fig. S6B), supporting this concept of common backbones that remain stable while allowing genes to be gained by insertion.

**Fig. 2:**
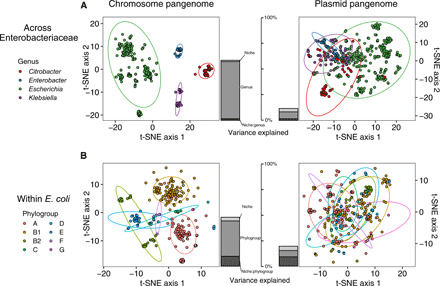
The plasmid-borne component of the pangenome is structured by niche and phylogeny, with greater variation than in the chromosomal component. Plots are shown for isolates (**A**) across Enterobacteriaceae and (**B**) within *E. coli*, for both the chromosomal component of the pangenome and the plasmid-borne component analyzed separately. Color indicates (A) genus within Enterobacteriaceae and (B) phylogroup within *E. coli*. Stacked bar charts in the center of each show the variance in gene content explained by niche, phylogeny (genus or phylogroup), and their interaction. The plasmid-borne component has greater residual variance than the chromosomal component, with a comparatively stronger niche-phylogeny interaction (darkest shaded bar).

A median of 3.3% of genes were on plasmids (range: 0 to 16.5%), with substantial variation by genus and niche (fig. S7A). This is a comparable value to the 3% finding of Touchon *et al.* (*30*) in *E. coli* with variation over a similar range [Fig. 4A of (*30*)]. We also considered the effect of plasmid copy number (i.e., multiplying plasmid lengths by their inferred copy number) to calculate the total DNA in both chromomes and plasmids within isolates (fig. S7B). *E. coli* isolates had a median of 5.7% of DNA present on plasmids, which was substantially higher in pig farm isolates (median: 10.1%; fig. S7B), linked to the presence of large plasmids. We analyzed the variation of gene content with phylogeny and niche (Fig. 2). Chromosomal genes were highly genus specific (*R*^2^ = 55.0%); the plasmid-borne pangenome was far more variable but still had a weak association with genus (*R*^2^ = 6.5%) (Fig. 2). Within *E. coli*, plasmid gene content was linked to niche (*R*^2^ = 5.6%) and phylogroup (*R*^2^ = 5.2%), with a stronger interaction between niche and phylogroup (*R*^2^ = 7.9%) (Fig. 2).

Plasmids were predicted to be conjugative, mobilizable, or nonmobilizable (see Materials and Methods), and we explored whether this affected their distribution. Nonmobilizable plasmid clusters were less commonly shared between different phylogroups within farms compared with mobilizable or conjugative plasmids (fig. S8). Although AMR genes were predominantly found in conjugative or mobilizable plasmid clusters, consistent with their frequent acquisition and movement between strains, plasmid clusters with AMR genes were not more commonly distributed across multiple phylogroups (chi-square test χ^2^ = 0.64, *P* = 0.42; fig. S8). However, on pig farms, most of the conjugative plasmid clusters seen across multiple phylogroups carried AMR genes, suggesting that they play an important role within this niche.

Since many isolates had multiple plasmids in their genome, we also considered the possibility of plasmid-plasmid interactions. Positive epistasis between large (>10 kbp) and small plasmids has been suggested to promote plasmid stability in Enterobacteriaceae, based on analyses of genomes in public databases (*32*). In *E. coli* isolates with complete genomes (*n* = 348), we observed a significant association between small and large plasmid presence (chi-square test χ^2^ = 4.44, *P* = 0.035), with 45.7% carrying at least one large (>10 kbp) and one small plasmid and only 3.7% carrying a small plasmid without a large plasmid. Thus, previous results that support the existence of positive large-small plasmid epistasis are reproducible in this natural population, suggesting that this is an important feature of Enterobacteriaceae plasmid distributions. We also found evidence for specific plasmid-plasmid associations. For example, cattle *E. coli* isolates showed co-occurrence of a ColRNA plasmid (cluster 37: median length, 4.6 kbp) and an IncFII plasmid cluster (cluster 279: median length, 106 kbp), with 14 of 16 isolates with the ColRNA plasmid also carrying the larger IncFII plasmid. Isolates were from three phylogroups (A: *n* = 2, B1: *n* = 5, and E: *n* = 9) and four farms, suggesting a robust association that reflects plasmid epistasis independent of chromosomal background.

### Plasmids carry an overrepresentation of AMR genes and insertion sequences

Plasmids carried more diverse and less genus-restricted genes, as expected from their role as the more flexible component of the genome. Despite carrying just 3.3% of total gene content, plasmid-borne genes accounted for 11.5% of unique genes (8.9 to 17.0% considering each genus; fig. S9), and 40.1% were seen in more than one genus (19.6 to 55.6% considering each genus; table S3). Plasmids are important vehicles of AMR genes in clinical contexts; similarly, plasmids had a much greater burden of AMR genes in the niches studied here. Considering isolates with circularized chromosomes (see Materials and Methods), 901 of 1876 AMR genes (48.0%) were found on plasmids, i.e., a 14.5× relative burden in plasmids. They also had a higher burden of insertion sequences (ISs), which are linked to the movement of genes by HGT. Of 26,565 ISs, 3695 (21.7%) were found on plasmids (6.6× relative burden). There was a weak correlation between the number of plasmid- and chromosome-associated AMR genes within an isolate (Spearman’s ρ = 0.11, *P* = 0.004) but a strong positive correlation for the number of ISs (Spearman’s ρ = 0.40, *P* < 0.001) (fig. S10A) seen across genera (fig. S10B).

We observed different patterns of ISs across chromosomes and plasmids (fig. S11). Some ISs were strongly associated with plasmids, with the strongest association being for IS*26*. However, 27.5% of isolates carrying IS*26* on a plasmid also carried it on their chromosome, consistent with its characteristically active behavior. It has been shown that IS*26* can drive the reorganization of plasmids in clinical settings by replicative transposition (*33*), as well as amplify AMR genes (*34*) and create within-plasmid heterogeneity in a single isolate (*35*). Its high prevalence in Enterobacteriaceae plasmids here suggests that it may play a similar role outside the context of clinical AMR. The most prevalent IS on both chromosomes and plasmids was IS*Kpn26*, with 50.2% of IS*Kpn26*-positive isolates having it both chromosomally and plasmid borne.

Considering *Escherichia*, WwTW isolates showed a greater diversity of ISs, with 65% of ISs found in a higher proportion of WwTW isolates compared with those from farms (fig. S12), including IS*30* which has been proposed as a marker for naturalized wastewater populations of *E. coli* (*36*). Touchon *et al.* (*30*) suggest that water-borne *E. coli* strains are adapted to this niche rather than being fecal contaminants and can therefore adapt by acquiring genetic material from not only gut bacteria (as in, e.g., a livestock or human host) but also other diverse environmental bacteria. The observation of greater IS diversity in WwTW isolates here would be consistent with this hypothesis.

We also investigated the overall patterns of co-occurrence of ISs to see whether they were strongly linked on plasmids. Overall, ISs had random levels of co-occurrence on *Escherichia* plasmids (upper tail *P* = 0.85 from null model simulations of checkerboard score; see Materials and Methods; fig. S13A), suggesting that ISs frequently move independently between plasmid backgrounds. In contrast, applying the same method to AMR genes, we found they significantly co-occurred (upper tail *P* = 0.02; fig. S13B), suggesting coselection and underlining the tendency of some AMR genes to co-occur in specific regions of plasmids.

### Plasmids carrying AMR genes show features suggestive of selection

**Fig. 3:**
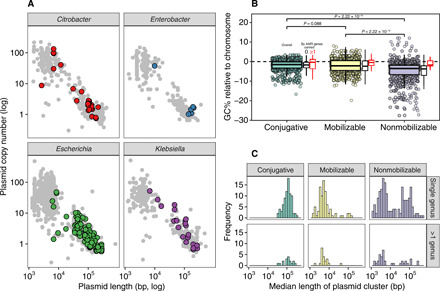
Distinct plasmid lifestyles between AMR and non-AMR plasmids. (**A**) Plasmid length (*x* axis) and inferred copy number (*y* axis) of all circularized plasmids (*n* = 2292), faceted by genus. Plasmids with ≥1 AMR gene (colored points) tended to be larger and present in lower copy numbers. (**B**) Relative GC content of all plasmids to their host chromosome for all circularized plasmids present in an assembly with a circularized chromosome (*n* = 1753 plasmids across 616 isolates), split by predicted plasmid mobility. Boxplots are additionally shown classifying plasmids within predicted mobility types by the number of AMR genes carried: those ≥1 AMR gene (red) or no AMR genes (black). Comparisons with *P* values are shown for all plasmids within a predicted mobility class. (**C**) Length distributions of plasmid clusters (see Materials and Methods).

Plasmids fell into two broad classes across genera: small multicopy plasmids (<10 kbp, 10× to 100× copy number inferred from coverage relative to chromosome) and large low-copy plasmids (>10 kbp, <10×) (Fig. 3A). AMR plasmids were almost all large low-copy plasmids (172 of 183, 94.0%). While small multicopy plasmids are of interest in facilitating evolutionary innovation (*37*, *38*), this finding suggests that they do not play a major, direct role in AMR in livestock- and WwTW-associated Enterobacteriaceae.

Experimental evidence shows that selective advantages favor lower GC content in intracellular elements (*39*), and this has been proposed as an explanation for the tendency of plasmids to typically have lower GC content than their host chromosome. Under this hypothesis, the more dependent on the bacterial host the plasmid is, the lower its relative GC content should be. We investigated this in our dataset. Overall, plasmids had a lower relative GC content than their host chromosomes (median difference, 2.5%; Fig. 3B). There was an approximate gradient of relative GC content with predicted plasmid mobility (Fig. 3B), with plasmids predicted to be mobile having a smaller relative difference. Furthermore, this difference was less marked for AMR plasmids (median, 0.3%) across mobility categories (Fig. 3B). Nearly half had a higher GC content than their host chromosome (46.7% versus 17.7% of non-AMR plasmids). Together, this suggests that AMR plasmids are being selected for in these environments, which counteracts the usual selective advantages for lower GC content. Alternatively, this may be a signal of their relatively recent acquisition by their host.

### Evidence for recent HGT across genera and within isolates

We identified 2364 potential HGT events involving transfers of sequence >5000 base pairs (bp) between isolates of different genera (see Materials and Methods). These represent possible instances of the recent movement of genetic material across species boundaries. Isolates from the same farm were ~10× more likely to show evidence of cross-genera HGT than would be expected (chi-square test χ^2^ = 1159, *P* < 0.001; fig. S14), and 12.3% of these cross-genera HGT events involved at least one AMR gene, with most of these AMR HGT events between pig isolates (37 of 48, 77.0%).

The movement of genes can also occur within individual genomes. We therefore also investigated occurrences where the same gene was present on both the chromosome and plasmid(s) within an *E. coli* genome. We observed distinct differences between niches, with increased amounts of chromosome-plasmid sharing in pig and WwTW isolates compared with cattle and sheep (fig. S15). This may be a signature of increased selection for AMR in these niches, such that usually transitory gene movements and duplications are retained in genomes for long enough to be detected.

### Quantifying the roles of phylogeny, niche, and geography in the *E. coli* pangenome

**Fig. 4:**
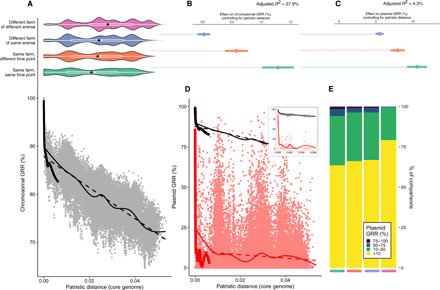
The interplay of phylogeny and niche in the *E. coli* pangenome. (**A**) Pairwise comparisons of GRR for chromosomal genes show that chromosomal GRR falls off rapidly at small patristic distances, followed by an approximately linear decrease. Fits show intra-ST comparisons (thick black line), all comparisons (thin black line), and a linear model (dashed black line). Violin plots above show the distribution of patristic distances depending on the relative sample source of the two isolates in the pairwise comparison (white boxplot: median and IQR; black point: mean), showing that even isolates cultured from the same sample (same farm and same TP) span equivalent diversity to isolates cultured from different locations. (**B**) Coefficients from a linear model for chromosomal GRR with an interaction term with patristic distance (excluding intra-ST comparisons). (**C**) Variance explained by phylogeny and geography for chromosomal and plasmid GRR. (**D**) GRR for plasmid-borne genes with patristic distance. Fits show intra-ST comparisons (thick red line), all comparisons (thin red line), and a linear model (dashed red line). Inset panel shows left-hand region of the plot with only intra-ST comparisons, with chromosomal GRR relationship also shown (gray points, black line). (**E**) Plasmid GRR comparisons shown by isolate sources, excluding intra-ST comparisons. Colors on the *x* axis are the same as in (A). Plots include all *E. coli* isolates with a circularized chromosome (*n* = 363).

To understand the strength of different factors shaping the pangenome, we analyzed the pangenome of a single species, *E. coli*, in more detail. Isolates recovered from the same location spanned total *E. coli* diversity (Fig. 4A). Interisolate core genome distances were strongly correlated with chromosomal gene repertoire relatedness (GRR) (Fig. 4A). Core genome distance explained the majority of variance in chromosomal GRR (Fig. 4B), but there was a consistent contribution from geography and time: isolates from the same pooled sample sharing more genes than would be expected (+1.2%), as did isolates from the same farm at different time points (+0.5%) (Fig. 4B). There was no such effect for isolates from different farms of the same livestock, suggesting that this reflects local geography rather than adaptation to livestock host. Although the variance explained was much lower, local geography effects were also observed for plasmid GRR (Fig. 4C), but core genome distance was uncorrelated with plasmid GRR apart from for near-identical strains (Fig. 4D). Isolates from different STs from different farms of the same livstock could still have high plasmid GRR (Fig. 4E), suggesting that host-specific plasmids may facilitate niche adaptation.

## DISCUSSION

We have investigated the pangenome of major genera of sympatric Enterobacteriaceae from locations within a 30-km radius, using a diverse set of nonclinical isolates cultured from the same samples and focusing in detail on *E. coli*. Despite high overall diversity, with most of the strains only observed once in the dataset, we observed the persistence of strains and plasmids on farms over the course of a year. Our results highlight the combination of persistence and dynamism that characterizes Enterobacteriaceae genomes at multiple scales, with relevance both for understanding the population structure of species within Enterobacteriaceae and for managing AMR. The existence of farm-level differences in *E. coli* populations that persists over time, with a small number of possible interfarm transfers, suggests that livestock farms function as distinct but linked niches. It could be that “everything is everywhere” (frequent movement of strains and genes between farms), but “the environment selects” (different farms have different selective pressures). However, the observation of persistent strains over the course of a year on farms, despite presumably varying selective conditions, and the overrepresentation of putative cross-genera HGT events in isolates at the same location suggest that geographical effects or intrinsic properties of certain bacterial/MGE lineages could affect the evolution of AMR on these time scales. Future modeling work and investigation will be required to distinguish these hypotheses. Overall, our findings underline the importance of local control strategies for the emergence and spread of AMR beyond clinical settings.

Here, we have focused on genome dynamics across different niches but have not attempted to integrate our findings with detailed contextual information from the participating sites. This will be crucial to give further insight into how differences in antimicrobial usage on farms and other management practices can affect AMR in multispecies pangenomes, such as the decline in *mcr-1* prevalence in Enterobacteriaceae on pig farms after a ban on colistin in feed, as observed in both China (*40*) and the UK (*41*). Other factors beyond antimicrobial usage could include herd size, herd management practices, and cleaning and disinfection practices. Investigating these is part of our ongoing work. Similarly, differences in management between WwTWs may affect the prevalence of AMR genes in effluent, and this is also the subject of ongoing work.

Resource limitations meant that we were unable to sequence and genetically evaluate all isolates that were cultured, and despite detailed sampling, we will not have captured all the persistence, HGT, and strain-sharing events across niches: The instances of sharing that we did identify remain fairly anecdotal. This is a problem faced by even intensive sampling efforts, which can only capture a tiny fraction of the population diversity and so are unlikely to retrieve close links in transmission chains (*42*) (of strains or MGEs). Future analyses could still investigate the links between such sharing and possible transmission routes between both farms and WwTWs, including the land application of manure (*43*), the land application of sewage sludge (*44*), and groundwater flow (*45*). Older studies have established that specific AMR genes can be more prevalent in groundwater closer to manure storage (*46*), but working out how to do equivalent genomic analyses for strains and MGEs across larger geographic distances with such diversity is difficult. Although this study is unprecented in evaluating four genera in such detail, AMR gene dissemination and important structural associations of AMR genes and MGEs may also be occurring within other genera not studied here. Furthermore, we did not investigate the relationship between isolates in this study and clinical human compartments in the same study area. Ludden *et al.* (*29*, *47*) previously reported limited direct overlap for *E. coli* or *Klebsiella pneumoniae* strains from livestock and humans in a different region of England but highlighted that 5% of human *E. coli* isolates potentially shared closely related AMR-associated MGEs with those found in livestock. We intend to see whether this finding holds in our ongoing work.

In conclusion, our study highlights the plastic and dynamic nature of AMR gene dissemination within the pangenome of major Enterobacteriaceae in several important nonclinical niches. It also demonstrates how robustly evaluating the flow of AMR genes and MGEs across highly diverse and dynamic niches is challenging even with extensive sampling. The implications of this for adequately understanding dissemination and selection of AMR genes in a “One Health” context should not be underestimated.

## MATERIALS AND METHODS

Isolates were sequenced from samples collected as part of the “The environmental REsistome: confluence of Human and Animal Biota in antibiotic resistance spread” (REHAB) study in 2017, which aimed to characterize nonclinical Enterobacteriaceae populations in four different niches within a defined study area of South Central England: cattle farms, pig farms, sheep farms, and water environments linked to WwTWs. Sampling occurred at each location at three separate TPs: January to April 2017 (TP1), June to July 2017 (TP2), and October to November 2017 (TP3).

### Farms

Five cattle farms, five sheep farms, and four pig farms were recruited from the study area following a defined recruitment process. Briefly, we aimed to recruit the five largest farms for each livestock type within the area using local APHA databases, progressively inviting the next largest farm if a farm declined. All participating farmers provided written consent for farm sampling for research purposes, and farm samples were taken between January and November 2017 on three separate visits (“TPs”) for each farm. Each farm was divided in five or fewer “epidemiological groups,” defined as a group of animals expected to share similar characteristics and managed in the same way. Ten pooled samples were collected from each of these groups, with each sample composed of small pinches of fresh feces from the floor combined into a small composite sample around 5 cm in diameter. Each group’s 10 samples were pooled, diluted up to 10^−5^ in phosphate buffer solution (pH 7.2), and plated onto CHROMagar ECC (CHROMagar Microbiology, Paris, France) and CHROMagar ECC plates containing cefotaxime (1 mg/liter) as a marker for multidrug resistance. Up to 10 colonies were collected from cefotaxime (1 mg/liter)–supplemented plates and 14 colonies from CHROMagar ECC plates; where 10 colonies were not recovered, additional colonies were taken from the CHROMagar ECC plates, resulting in 24 isolates per farm. Pure isolate subcultures were subsequently stored at −80°C in MicroBank beads (Pro-Lab Diagnostics, Neston, Cheshire, UK), and the bacterial species were confirmed using matrix-assisted laser desorption/ionization time-of-flight (MALDI-TOF) (Bruker, Coventry, UK) or 16*S* rRNA sequencing (*48*). The median number of sequenced isolates for a farm TP combination was 12 (range: 9 to 14), with 495 farm isolates in total: cattle (*n =* 178), pig (*n* = 143), and sheep (*n* = 174).

### Wastewater treatment works

Five WwTWs were selected on the basis of a defined recruitment process including the following: geographic location within the study area, wastewater treatment configuration, wastewater population equivalent served, consented flow, and the accessibility of the effluent receiving river for sampling both upstream and downstream. Sampling took place in 2017 over three sampling rounds: February to March (TP1), June to July (TP2), and October to November (TP3). Sewage influent samples were collected after WwTW coarse screens, and effluent samples were collected at the last sampling point before entering the river. For each sampling round, ~6 repeated 200-ml samples of influent and effluent were collected between 9:00 a.m. and 12:00 p.m. using a sampling pole and sterile Whirl-Pak collection bags. Repeat samples in each round were pooled before processing to reduce the impact of temporal variability in wastewater flows and composition. Sediment samples were collected from 100 m upstream and 250 m downstream of the effluent entry point into the river. Sediment samples were collected using a custom sampling pole that held a removable 50-ml plastic centrifuge tube (Sigma-Aldrich, UK). Using a fresh sterile 50-ml tube each time, sediment from the riverbed was collected from the surface layer at three points at each sampling site; near bank, the center of the river, and the far bank. These samples were pooled before analysis to account for spatial variability in sediment composition. Influent, effluent, and sediment samples were stored in an insulated box at ~4°C until getting back to the laboratory (<6 hours). Influent, effluent, 100 m upstream, and 250 m downstream environmental samples collected from each sewage treatment works were transferred to the laboratory on ice and processed within 24 hours of collection. Each sample was vortexed briefly, serial diluted to 10^−3^ in nutrient broth containing 10% glycerol (Oxoid, Basingstoke, UK), and plated onto CHROMagar Orientation agar (Chromagar, Paris, France) and CHROMagar Orientation agar supplemented with cefotaxime (1 μg/ml) (Cambridge Biosciences, Cambridge, UK). Colonies with putative morphology for species of interest were subcultured from dilution plates with suitably isolated growth. A total of up to 20 colonies was picked per sample: Up to 10 colonies were picked from the cefotaxime (1 mg/liter)–supplemented plates, and the remainder were picked from the nonsupplemented plates. Pure isolates subcultured on Columbia blood agar (CBA) (Oxoid, Basingstoke, UK) were subsequently stored at −80°C in nutrient broth containing 10% glycerol, and bacterial species were confirmed using MALDI-TOF (Bruker, Coventry, UK).

### DNA sequencing

A subset of isolates were selected for sequencing to represent diversity within the four major genera within each niche, including the use of third-generation cephalosporin resistance as a selective marker to identify a subgroup of multidrug-resistant isolates within each genus. A total of 832 isolates were each sequenced with both a short-read (Illumina HiSeq 4000) and a long-read sequencing approach [four isolates selected for sequencing failed subsequent hybrid assembly and were not included in further analyses; one further isolate was removed after assembly (see “Genome assembly”)]. For the first TP, the latter involved sequencing using either PacBio SMRT (*n* = 192) or Oxford Nanopore Technologies (ONT) methodologies (*n =* 127). The results of a pilot study comparing sequencing and assembly approaches using a subset of REHAB isolates (*28*) were used to inform the choice of ONT as the long-read sequencing approach for all isolates from the second (*n* = 255) and third (*n* = 254) TPs.

Isolate stocks from −80°C storage were cultured onto CBA and supplemented with cefpodoxime (Thermo Fisher Scientific, USA) 10-μg discs for isolates not sensitive to cefotaxime during original sample isolation. DNA was extracted using the Qiagen Genomic tip/100G (Qiagen, Venlo, The Netherlands) according to the manufacturer’s instructions. DNA concentration was quantified by Qubit 2.0 fluorimeter (Invitrogen, UK), and quality and fragment size distribution were assessed by TapeStation 2200 (Agilent, Santa Clara, USA). ONT sequencing libraries were prepared by multiplexing six to eight DNA extracts per flow cell using kits SQK-RBK004, SQK-LSK108, and EXP-NBD103 according to the manufacturer’s protocol. Libraries were loaded onto flow cell versions FLO-MIN106 R9.4 (0.1) SpotON and sequenced for 48 hours on a GridION (ONT, Oxford, UK).

### Genome assembly

We used the hybrid assembly and sequencing methods described in our pilot study (*28*) to produce high-quality Enterobacteriaceae genomes from short and long reads. Briefly, we used Unicycler (v0.4.7) (*49*) with “normal” mode, --min_component_size 500, --min_dead_end_size 500, and otherwise default parameters. Our pilot study (*28*) explored the accuracy of assemblies from this method (and others) in detail using multiple metrics, including the following: the mapping of Illumina short reads back to assemblies, the mapping of long reads back to assemblies, the comparison of assemblies from the same isolate, using known marker genes to assess overall quality, the assembly’s overall “circularity,” and the presence of indel errors. We found excellent concordance in structural agreement of circular structures from different assemblies [see, e.g., fig. S4 of (*28*)], giving us confidence that circular structures from hybrid assemblies (chromosomes and plasmids) are accurate. One possible concern could be that chimeric reads in long-read datasets could lead to incorrect structures in the final hybrid assembly. However, an independent investigation (*50*, *51*) using data from our pilot study alongside simulated data showed that long read–only assemblies with Unicycler are only affected by the presence of chimeric reads at rates of ~15% of reads and are thus not a concern for real datasets, which typically have rates of <2%.

Final assemblies from all isolates had a median of four contigs [interquartile range (IQR): 3 to 8; range: 1 to 391], with a median of two circularised plasmids (IQR: 1 to 4, range: 0 to 14). One *Citrobacter* isolate from TP1 was removed from the dataset after we identified a sample mixup, meaning that its metadata were unreliable. The majority (616 of 827, 74.5%) of the assemblies had a circularized chromosome, and 558 of 827 (67.4%) were complete, i.e., chromosome and all plasmids circularized (table S1).

### Genome assignment and typing

We assigned species and ST from assembled genomes using mlst (v2.16.4) (*52*). We also validated species assignments by downloading all National Center for Biotechnology Information (NCBI) Refseq complete genomes for the four genera under study as of 4 June 2020 and using fastANI (v1.3) (*53*) to compute average nucleotide identity scores against reference genomes for each assembled genome. We took the species assignment of the top hit for each assembled genome. Furthermore, we manually checked genus assignments using a t-distributed stochastic neighbor embedding (t-SNE) plot of isolate genomes against a collection of reference genomes and made corrections to the assignment if necessary. We used ClermonTyping (v1.4.1) (*54*) to assign phylogroup to *n =* 553 *Escherichia* isolates. Considering the genus *Escherichia*, there were 553 isolates, 410 with circularized chromosomes, and of these, 379 were complete genomes containing 961 complete plasmids in total. Considering only *E. coli*, there were 502 *E. coli* isolates, 372 with circularized chromosomes, and of these, 348 were complete genomes containing 878 complete plasmids in total. A minority of genomes were *E. fergusonii* (*n* = 51), from clades I to V (*n* = 14), or could not be typed (*n* = 7), with *n* = 481 genomes from within the principal *E. coli* phylogroups (A: *n* = 131; B1: *n* = 193; B2: *n* = 59; C: *n* = 11; D: *n* = 25; E: *n* = 50; F: *n* = 6; and G: *n* = 6).

Sequenced isolates from three other Enterobacteriaceae genera included the following: *Citrobacter* (*n* = 128: 81 *Citrobacter freundii* and 46 unassigned *Citrobacter* sp.), *Enterobacter* (*n* = 71: 59 *Enterobacter cloacae* and 12 unassigned *Enterobacter* sp.), and *Klebsiella* (*n* = 76: 40 *K. pneumoniae*, 30 *Klebsiella oxytoca*, 2 *Klebsiella aerogenes*, and 4 unassigned *Klebsiella* sp.). The majority of the farm-associated isolates were *E. coli*, whereas WwTW-associated isolates had roughly equal numbers of genera (fig. S1). This reflects both the diversity present in each niche and the selection strategy to sequence equal numbers across genera where feasible.

### Pangenome analysis

All genomes were annotated with Prokka (v1.14.0) (*55*). We performed a multispecies pangenome analysis by clustering genes into gene groups using Roary (v3.12.0) (*56*) across all isolates at various sequence identity thresholds with the maximum number of clusters set to 300,000 (-g 300,000) and without splitting paralogs (-s). At a 95% identity for blastp, there were 139,788 gene groups across all genera. Further to this analysis, genes were also clustered at a higher sequence identity (>99% identity threshold) to identify recent HGT events, which gave 214,743 gene groups across all genera. (These pangenome analyses included the subsequently removed *Citrobacter* isolate with unreliable metadata.) For *n* = 616 isolates with circularised chromosomes, we split the genome into chromosomal and plasmid-borne components (i.e., all other contigs) to analyze the genomic location of genes. We excluded isolates without circularized chromosomes from this analysis. For within-species pangenome analyses, the more recently developed Panaroo gives lower annotation error rates and a more accurate core genome than Roary or other methods by using gene adjacency (i.e., synteny) information (*57*), although it is not suitable for cross-species analyses where no core genome is expected. Therefore, for a higher-resolution within-species analysis of *n* = 488 *E. coli* isolates (excluding *E. fergusonii* and clades I to V), we used Panaroo (v0.1.0) (*57*) to extract a core genome alignment based on 2915 concatenated core genes (Fig. 1C). The phylogeny was produced using iqtree (v1.6.11) (*58*), with branch lengths not corrected for recombination, and plotted with ggtree (v2.0.1) (*59*).

### Plasmid annotation and clustering

We searched all plasmids against PLSDB (version: 2020-03-04) (*60*), which contains 20,668 complete published plasmids, using “screen” in mash (v2.0) (*61*) and keeping the top hit. All plasmids had a match apart from 17 small plasmids predicted to be nonmobilizable (median length: 4.8 kbp; range: 2.9 to 20.7 kbp), from *Escherichia* (*n* = 11), *Enterobacter* (*n* = 2), and *Citrobacter* (*n* = 4). We clustered plasmids using mob cluster and assigned replicon types with mob typer, both part of the MOB suite (v1.4.9) (*62*). Mob cluster uses single linkage clustering with a cutoff of a mash distance of 0.05 [corresponding to 95% average nucleotide identify (ANI)], resulting in 611 clusters (table S2). In total, there were 134 different combinations of replicons observed on plasmids (“replicon haplotypes”). The most abundant replicon was IncFIB (*n* = 459), which was seen across all niches [pig (*n* = 80), cattle (*n* = 113), sheep (*n* = 78), and WwTWs (*n* = 188)]. Only nine small multicopy plasmids (~6 kbp) carried AMR genes, all of which had a ColRNAI replicon; these ColRNAI plasmids have been proposed to be sources of evolutionary innovation (*37*, *38*).

We considered the relationship between such “distance-free” clustering and plasmid gene content. On the basis of gene clustering with Roary (see above), we compared the structure of circularised plasmids using all connecting edges between two genes. We defined the resemblance for both gene content (gene presence/absence) and gene structure. The gene content resemblance between two plasmids with *n*_1_ and *n*_2_ genes, respectively, with *N* genes in common, was defined as *r*_content_ = 2 *N*/(*n*_1_ + *n*_2_). The edge structure resemblance between two plasmids with *g* gene-gene edges in common was defined as *r*_edge_ = 2 *g*/(*n*_1_ + *n*_2_). Typically *r*_edge_ < *r*_content_, but this definition does allow for the case where repeated genetic elements produce *r*_edge_ > *r*_content_ (e.g., fig. S6B).

### Comparison of plasmid-borne and chromosomal pangenome components

To visualize cross-genera pangenomes (e.g., Fig. 2), we used t-SNE. We used the Rtsne function with a perplexity of 30 on gene presence/absence matrices in the Rtsne R package (v0.15) (*63*). To conduct permutational analyses of variance, we used the adonis function from the vegan R package (v2.5-6) (*64*) on the matrix of pairwise Jaccard distances, which was calculated using the vegdist function. For between-genera analyses, we used the formula dist~niche*genus. For within-*Escherichia* analyses, we used the formula dist~niche*phylogroup.

### Detection of AMR genes and ISs

We searched assemblies using ABRicate (v0.9.8) (*65*) for acquired resistance genes (i.e., excluding mutational resistance) in the NCBI AMRFinder Plus database (PRJNA313047). We used a minimum identity threshold of 90% and a minimum coverage threshold of 90% (table S4). Isolates that cultured selectively from cefotaxime-supplemented plates carried more AMR genes than nonselectively cultured isolates (median of 7.5 versus 1.0), as expected. We also searched for ISs using the ISFinder database (*66*) as a database in ABRicate with the same identity and coverage thresholds (table S5).

### Detection of recent HGT events

We performed an all-against-all comparison of assemblies with mummer (v3.23-2) (*67*) using the -maxmatch option to identify shared sequences of length >5000 bp between genomes of different genera (these could include both transfer of whole plasmids or partial sequences). For comparing the observed distribution of cross-genera HGT events to the expected, we assumed a random distribution drawn from all possible cross-genera comparisons from livestock isolates.

### Distribution of ISs

We constructed the bipartite presence/absence network of ISs and replicon haplotypes for the 34 replicon haplotypes, which were observed on 10 or more plasmids. We simulated null models of co-occurrence patterns using the cooc_null_model with null model sim9, which fixes the row and column sums of the presence/absence matrix, in the R package EcoSimR (v0.1.0) (*68*). Simulations used *n* = 10,000 iterations with a burn-in of 500 iterations.

### Modeling of GRR

We selected a subset of *E. coli* genomes with a circularized chromosome (*n* = 363) and used the core genome tree constructed with iqtree (Fig. 1; dropping other *E. coli* isolates) to calculate patristic distances between isolates. We calculated chromosomal and plasmid GRR for all pairwise comparisons using output from roary (95% identity threshold, as above) and fit linear models for GRR (Fig. 4).

## SUPPLEMENTARY MATERIALS

Supplementary material for this article is available at http://advances.sciencemag.org/cgi/content/full/7/15/eabe3868/DC1
